# *Helicobacter pylori* Infection: Clinical, Endoscopic, and Histological Findings in Lebanese Pediatric Patients

**DOI:** 10.1155/2020/4648167

**Published:** 2020-05-11

**Authors:** F. AL Kirdy, M. Rajab, N. El-Rifai

**Affiliations:** Department of Pediatrics, Makassed General Hospital, Beirut, Lebanon

## Abstract

**Background:**

*Helicobacter pylori* (*H. pylori*) is a common and universally distributed bacterial infection. However, in children, active gastritis and ulcer are rarely seen.

**Objectives:**

The aims of this study were to establish the prevalence of *H. pylori* infection and to compare the clinical, endoscopic, and histopathological findings between infected and noninfected pediatric patients at Makassed General Hospital.

**Methods:**

Patients aged between 1 month and 17 years who underwent upper gastrointestinal endoscopy from January 2011 to January 2017 were included. The diagnosis of *H. pylori* was confirmed by a CLO test and/or its presence on biopsy specimens. Demographic data, clinical characteristics, endoscopic and histopathological findings, and gastritis score were recorded retrospectively.

**Results:**

During the study period, 651 children underwent upper gastrointestinal endoscopy. The main indication was abdominal pain (61%). The prevalence of *H. pylori* infection was 16.5%. The infection was most commonly seen among children aged between 6 and 10 years (43%). A large number of family members were associated with increased risk of infection (4.8 ± 1.5 versus 5.2 ± 1.8; *p* < 0.05). Epigastric pain was more associated with *H. pylori* (61.3% versus 14.6% in noninfected patients; *p* < 0.05). Nodular gastritis was commonly seen in infected patients (41.5% vs. 7.9%; *p* < 0.05). Mild and moderate gastritis was seen more in infected versus noninfected patients (mild: 53.8% vs. 14%; moderate: 27.4% vs. 2.4%, respectively).

**Conclusion:**

Although epigastric pain was associated with *H. pylori*, other diagnoses should be considered since the infection are rarely symptomatic in children. Antral nodularity was associated with *H. pylori infection*; however, its absence does not preclude the diagnosis.

## 1. Introduction

*Helicobacter pylori* (*H. pylori*) is one of the most common and universally distributed bacterial infection. It was identified in gastric biopsies of gastritis patients by Warren and Marshall in 1983 [1]. It is acquired predominantly in childhood and may persist throughout life [2]. The rate of *H. pylori* infection is intimately correlated to the age, race, family members, and country's socioeconomic level. A higher incidence is found in patients aged 5 to 10 years, high family members, and developing countries [3]. In adults, *H. pylori* may lead to gastroduodenal ulcer or gastric cancer. However, these findings are rarely seen in children [4]. Recent data showed that *H. pylori* rarely give rise to symptoms in children in the absence of peptic ulcer disease (PUD) [5]. No correlation was found between recurrent abdominal pain and *H. pylori* infection [6]. However, there is conflicting data regarding the link between upper abdominal pain and *H. pylori* infection [6]

Although nodular gastritis predicts *H. pylori* infection and histologic gastritis, its specificity remains questionable [7]. In addition, the inflammatory response in children is different from that seen in adults. For these reasons, the most reliable method of diagnosis of *H. pylori* infection according to ESPGHAN and NASPGHAN guidelines is either histopathological identification of the bacterium with at least one other positive biopsy-based test (CLO test, PCR, or FISH) or positive culture [8].

The aim of this study was to investigate the clinical, endoscopic, and histopathological findings in *H. pylori*-infected and noninfected pediatric patients.

## 2. Methods

This was a retrospective cross-sectional study, held at the Pediatrics Department of Makassed General Hospital over a 6-year period between January 2011 and January 2017.

Patients aged from one month to 17 years who underwent upper gastrointestinal endoscopy for gastrointestinal symptoms, failed to thrive, and/or have short stature were included in the study. Patients who received H2 blockers, antimicrobials, or proton pump inhibitors (PPI) within 2-30 days of the procedure were excluded from data analysis.

Data regarding the age, sex, anthropometric measures, place of residence, number of family members, indications of gastroscopy, and family history of PUD or gastric cancer were recorded. The endoscopic and histopathological findings were collected from the medical records.

The materiel used for endoscopy was (Pentax®) video gastroscope (EG-2490K) (outer diameter: 8 mm). The examination was performed in the operating room under deep sedation or anesthesia. The possible endoscopic diagnosis included normal, superficial gastritis, nodular gastritis, and peptic ulcer disease. The endoscopic aspect of nodular gastritis was based on the irregular appearance of the mucosa as a cobblestone pavement.

Five gastric biopsy specimens were collected from each patient: two from the gastric antrum (one from the distal lesser curvature and one from the distal greater curvature), two from the corpus (from the greater curvature), and one from the area next to the incisura angularis for use in the CLO™ test (campylobacter-like organism test) on which the specimens were sent for histopathological analysis. No specimen was taken for culture since it is not available in our country. Biopsy specimens were fixed in 10% formalin embedded in paraffin and were stained with hematoxylin-eosin and modified Giemsa coloration technique. The same pathologist interpreted the biopsy results during the study period. When the CLO™ test showed red-violet color within 24 hours at room temperature and/or *H. pylori* was found on histopathologic examination in biopsy specimens, the diagnosis of H *pylori* infection was made.

The histologic findings were used to classify the *H. pylori* density and the type of inflammation. The inflammation was divided into monocyte infiltration, neutrophil infiltration, glandular atrophy, or intestinal metaplasia and scored as mild, moderate, or severe using the visual analogue scale applied to microscopic examination results according to updated Sydney system [9]. The sum of the scores obtained from each patient was used as the gastritis score.

The study was approved by the IRB. The Statistical Package for Social Sciences (SPSS, version 24) program was used for data analysis. Bivariate analysis was carried out by using the chi-square for comparing categorical variables. Student's *t*-test was used to compare continuous variables. A multivariate analysis was conducted to control for confounding variables. A *p* value ≤ 0.05 was considered significant.

## 3. Results

During the study period, 651 patients underwent upper gastrointestinal endoscopy. Ten of them were excluded for prior use of H2 blockers, PPI, and antibiotics. Abdominal pain was the most common indication for upper gastrointestinal endoscopy during the study period (67%), followed by failure to thrive (37.7%), suspected celiac disease (30.2%), short stature (25.5%), recurrent vomiting (16%), chronic constipation (7.6%), and hematemesis (2.8%). There was no statistical significance between *H. pylori* positive and negative patients concerning the indications of upper gastrointestinal endoscopy.

**Figure 1:**
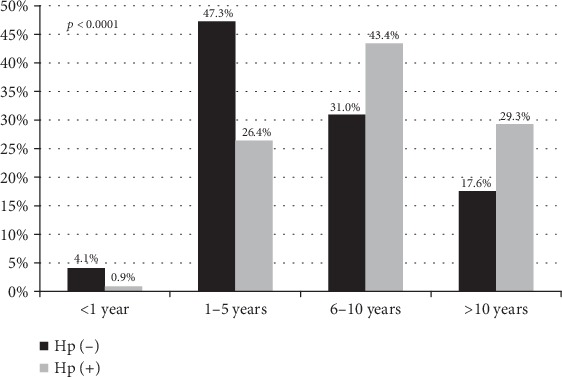
Age distribution in *Helicobacter pylori-*infected and noninfected patients. Hp(+): *H. pylori*-infected patients; Hp(-): *H. pylori* noninfected patients

The overall prevalence of *H pylori* infection was 16.5% (106/641). The CLO test was positive in 99/106 of *H. pylori*-positive patients (93.4%).  Figure 1 illustrates the distribution of the infection according to age. The highest prevalence was seen in patients aged between 6 and 10 years compared to other age categories with a significant *p* value (*p* < 0.0001). There was no significant difference between *H. pylori*-negative (Hp(-)) and *H. pylori*-positive (Hp(+)) groups concerning gender (*p* = 0.35). Higher weight (28 ± 14 vs. 21.6 ± 13.5 kg) and height (122.6 ± 20.6 vs. 108.5 ± 27.6 cm) were seen in *H. pylori*-positive versus *H. pylori*-negative patients, respectively, with a statistical significance (*p* < 0.0001). A higher number of family members were noted in *H. pylori*-positive patients versus *H. pylori*-negative patients (5.2 ± 1.8 versus 4.8 ± 1.5, respectively) with a statistical significance (*p* = 0.02). There was no significant difference between the two groups regarding the place of residence, the educational level of the guardian, the family history, and the socioeconomic level.

According to the characteristics of abdominal pain, diffuse abdominal pain was seen most commonly in *H. pylori*-negative patients versus *H. pylori*-positive patients (46.5% versus 5.7%, *p* < 0.0001). However, epigastric pain was seen most frequently in *H. pylori*-infected patients (61.3% versus 14.6%, *p* < 0.0001). But the frequency and duration of epigastric pain did not differ between the two groups.

**Figure 2:**
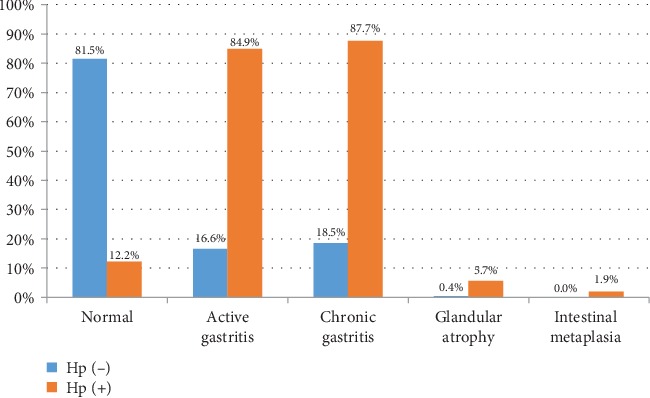
Histopathological findings in *Helicobacter pylori*-infected and noninfected patients.

**Figure 3:**
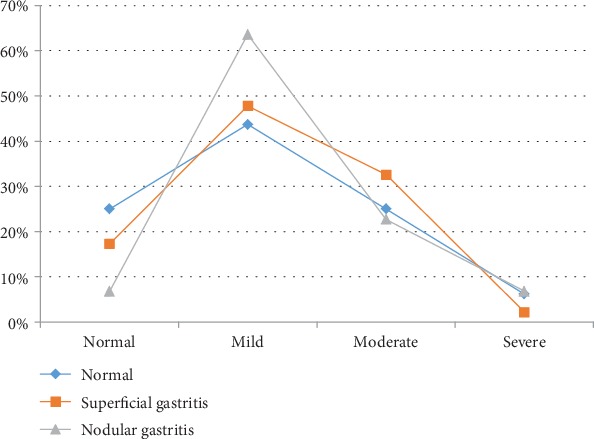
Correlation between the endoscopic findings and the gastritis score in infected patients.

Superficial and nodular gastritis were the most common endoscopic finding in *H. pylori*-positive patients (43.3% and 41.5%, respectively). In comparison, the gastric mucosa was normal in 44.8% of *H. pylori*-negative patients with superficial gastritis in 46.3% of them. No peptic ulcer disease was seen in infected patients.  Figure 2 illustrates the histopathological findings of *H. pylori*-infected and noninfected patients. Chronic and active gastritis were the most common findings in *H. pylori*-infected patients (87.7% and 84.9%, respectively). However, glandular atrophy and intestinal metaplasia were rarely seen in both groups. As for the gastritis score, mild and moderate scores were seen in *H. pylori*-infected patients (53.8% and 27.4%, respectively). However, mild gastritis was seen in (14%) of *H. pylori*-negative patients. Severe degree of gastritis was seen only in 4.7% of infected patients.  Figure 3 illustrates the correlation between the gastritis score and the macroscopic endoscopic findings in infected patients. Nodular gastritis was most commonly associated with mild degree of gastritis.

## 4. Discussion

To the best of our knowledge, our study is the first one done in Lebanon reporting the prevalence of *H. pylori* infection in a pediatric age group based on invasive testing. Naous et al. in 2007 reported a fecoprevalence of *H. pylori* infection in asymptomatic Lebanese pediatric patients of 21% [10]. But according to the recent recommendations of ESPGHAN/NASPGHAN, the stool antigen test for *H. pylori* is used for the confirmation of eradication of the pathogen after the treatment and not as a diagnostic tool [8].

The prevalence of *H. pylori* infection varies worldwide ranging between 5 and 21% and reaching 79.4% in Bahrain [11]. In our study, the prevalence of *H. pylori* infection was comparable to those reported in the literature. The highest prevalence was seen among children aged between 6 and 10 years and decreased thereafter. This finding was supported by Rowland et al. and Sykora et al. who reported an increased risk of *H. pylori* in the same age group [12, 13]. Similarly, Alborzi et al. reported a significant reduction in the incidence of *H. pylori* infection in children more than 15 years [14]. This could be related to the lower sensitivity of the CLO test in younger ages due to lower bacterial density in children compared to adults [8].

In our study, the prevalence of *H. pylori* infection was comparable in boys and girls. Similarly, Martel and Parsonnet in their meta-analysis on 10 studies showed that there is no significant relationship between sex and incidence of *H. pylori* infection [15]. Surprisingly, we found relatively higher weight and height among *H. pylori*-infected children. This is supported by the lack of evidence that *H. pylori* infection could alter the growth in children [16–19].

The risk factors of *H. pylori* infection include poor socioeconomic conditions, family overcrowding, child care attendance, poor hygiene, and living with an infected family member and are higher in developing countries than in developed countries [3]. In our study, *H. pylori* infection was associated with a higher number of family members with poor socioeconomic status.

Abdominal pain was the main indication of upper gastrointestinal endoscopy in our study. In addition, there was a statistical significant association between epigastric pain and *H. pylori* infection, which was also reported by Yang et al. and Ng et al. [20, 21]. This mirrors the recent recommendation of the ESPGHAN/NASPGHAN for investigating patients for *H. pylori* infection with persistent upper gastrointestinal symptoms [8]. However, the infection was less prevalent in patients with diffuse abdominal pain.

Antral nodularity may be a pathognomonic macroscopic finding of childhood *H. pylori* infection [22]. The positive predictive value of nodular gastritis is variable in the literature ranging between 46% and 73% [7, 23].

In our study, superficial and nodular gastritis were most commonly seen among *H. pylori*-infected children (43.4%, 41.5%, respectively). Similarly, Luzza et al. found a high prevalence of antral nodularity in *H. pylori*-infected children followed by superficial gastritis (40 and 13%, respectively). Erosions and ulcers were present in 1% of *H. pylori*-positive patients [24]. Thus, the absence of nodular gastritis does not preclude the diagnosis of *H. pylori* which necessitates the identification of the bacterium histopathologically. In our study, none of the *H. pylori*-infected patients developed an ulcer disease.

Histologically, Mazigh Mrad et al. found that infection with *H. pylori* was significantly associated with chronic and active gastritis (88.5% and 63%, respectively) [25]. Similarly, in our study, chronic and active gastritis were seen in most of *H. pylori*-infected patients (87.7% and 84.9%, respectively). At the same time, *H. pylori* infection may be associated with normal appearing mucosa as reported by Cárdenas-Mondragón et al. [26]. In our study, 12.2% of infected patients had normal gastric mucosa on histopathology.

According to gastritis score, mild and moderate gastritis were seen more commonly in *H. pylori*-infected children than severe gastritis (53.8% and 27.4% versus 4.7%, respectively). Our results were comparable to those reported by Cárdenas-Mondragón et al. who found a mild to moderate degree of gastritis in *H. pylori*-infected patients compared to severe one (31% and 14.9% versus 4%, respectively) [26]. This could be explained by the less severe inflammatory response in children compared to adults. Koh et al. revealed that gastritis score was the only significant factor influencing the occurrence of nodular gastritis [27]. This was similar to our study, on which antral nodularity was most common with mild degree of gastritis. Anyone would expect that nodular gastritis occurs with severe gastritis score rather than mild, but the type of *H. pylori* immune reaction in children is completely different from adults due to the presence of T-regulatory cells which downregulates the inflammation and ulceration induced by *H. pylori* in children [28].

## 5. Conclusion

In our study, we found a positive correlation between epigastric pain and *H. pylori* infection. This highlights the importance of invasive testing of this pathogen in patients with persistent upper abdominal symptoms. Although antral nodularity was related to the histopathological presence of *H. pylori*, its absence does not exclude the diagnosis. As a result, the combination of the clinical, endoscopic, and histopathological findings remains crucial for a definitive diagnosis of *H. pylori* infection.

## Data Availability

The data used to support the findings of this study are available from the corresponding author upon request.

## References

[1] Warren J. R., Marshall B. (1983). Unidentified curved bacilli on gastric epithelium in active chronic gastritis. *The Lancet*.

[2] Mégraud F. (2010). Epidemiology of *Helicobacter pylori* infection. *Gatsroenterol Clin North Am*.

[3] Rosenberg J. J., Adam H. M. (2010). *Helicobacter pylori*. *Pediatrics in Review*.

[4] Gottrand F., Cullu F., Turck D. (1997). Normal gastric histology in *Helicobacter pylori*-infected children. *Journal of Pediatric Gastroenterology & Nutrition*.

[5] Sierra M. S., Hastings E. V., Goodman K. J. (2013). What do we know about benefits of *H pylori* treatment in childhood?. *Gut Microbes*.

[6] Spee L. A., Madderom M. B., Pijpers M., Van Leeuwen Y., Berger M. Y. (2010). Association between *Helicobacter pylori* and gastrointestinal symptoms in children. *Pediatrics*.

[7] Prasad K. K., Thapa B. R., Sharma A. K., Nain C. K., Singh K. (2008). Reassessment of diagnostic value of antral nodularity for *Helicobacter pylori* infection in children. *Minerva Gastroenterologica e Dietologica*.

[8] Jones N. L., Koletzko S., Goodman K. (2017). Joint ESPGHAN/NASPGHAN guidelines for the management of *Helicobacter pylori* in children and adolescents (update 2016). *Journal of Pediatric Gastroenterology and Nutrition*.

[9] Dixon M. F., Genta R. M., Yardley J. H., Correa P. (1996). Classification and grading of Gastritis. *The American Journal of Surgical Pathology*.

[10] Naous A., Al-Tannir M., Naja Z., Ziade F., El-Rajab M. (2007). Fecoprevalence and determinants of *Helicobacter pylori* infection among asymptomatic children in Lebanon. *Le Journal Médical Libanais*.

[11] Fakhro A. R., Fateha Bel D., Amin Farid I. M., Jamsheer H. M. (1999). The association between Helicobacter pylori infection and lymphoid reaction in patients suffering from dyspepsia in Bahrain. *Saudi Journal of Gastroenterology*.

[12] Rowland M., Daly L., Vaughan M., Higgins A., Bourke B., Drumm B. (2006). Age-specific incidence of *Helicobacter pylori*. *Gastroenterology*.

[13] Sýkora J., Siala K., Varvařovská J., Pazdiora P., Pomahačová R., Huml M. (2009). Epidemiology of *Helicobacter pylori* infection in asymptomatic children: a prospective population-based study from the Czech Republic. Application of a monoclonal-based antigen-in-stool enzyme immunoassay. *Helicobacter*.

[14] Alborzi A., Soltani J., Pourabbas B. (2006). Prevalence of *Helicobacter pylori* infection in children (south of Iran). *Diagnostic Microbiology and Infectious Disease*.

[15] De Martel C., Parsonnet J. (2006). *Helicobacter pylori* infection and gender: a meta-analysis of population-based prevalence surveys. *Digestive Diseases and Sciences*.

[16] Vilchis J., Duque X., Mera R. (2009). Association of *Helicobacter pylori* infection and height of Mexican children of low socioeconomic level attending boarding schools. *The American Journal of Tropical Medicine and Hygiene*.

[17] Gulcan M., Ozen A., Karatepe H. O., Gulcu D., Vitrinel A. Impact of *H pylori* on growth: is the infection or mucosal disease related to growth impairment?. *Digestive Diseases and Sciences*.

[18] Ozen A., Furman A., Berber M. (2011). The effect of *Helicobacter pylori* and economic status on growth parameters and leptin, ghrelin, and insulin-like growth factor (IGF)-I concentrations in children. *Helicobacter*.

[19] Yang Y. J., Sheu B. S., Yang H. B., Lu C. C., Chuang C. C. (2012). Eradication of *Helicobacter pylori* increases childhood growth and serum acylated ghrelin levels. *World Journal of Gastroenterology*.

[20] Yang Y. J., Sheu B. S., Lee S. C., Wu J. J. (2005). Short-term recurrent abdominal pain related to *Helicobacter pylori* infection in children. *Journal of Gastroenterology and Hepatology*.

[21] Ng B. L., Quak S. H., Aw M., Goh K. T., Ho B. (2003). Immune responses to differentiated forms of *Helicobacter pylori* in children with epigastric pain. *Clin Diagn Lab Immunol*.

[22] Yang H. R. (2016). Updates on the diagnosis of *Helicobacter pylori* infection in children: what are the differences between adults and children. *Pediatric Gastroenterology, Hepatology & Nutrition*.

[23] Sani M. N., Kianifar H. R., Khodadad A., Ahmadi M., Falsafi T., Khatami G. R. (2005). Endoscopic nodular gastritis: an indicator of *H pylori* infection in children. *MJIRI*.

[24] Luzza F., Pensabene L., Imeneo M. (2001). Antral nodularity identifies children infected with *Helicobacter pylori* with higher grades of gastric inflammation. *Gastrointestinal Endoscopy*.

[25] Mazigh Mrad S., Abidi K., Brini I., Boukthir S., Sammoud A. (2012). Nodular gastritis: an endoscopic indicator of *Helicobacter Pylori* infection in children. *La Tunisie Médicale*.

[26] Cárdenas-Mondragón M. G., Carreón-Talavera R., Camorlinga-Ponce M., Gomez-Delgado A., Torres J., Fuentes-Pananá E. M. (2013). Epstein Barr virus and *Helicobacter pylori* co-infection are positively associated with severe gastritis in pediatric patients. *PLoS One*.

[27] Koh H., Noh T. W., Baek S. Y., Chung K. S. (2007). Nodular gastritis and pathologic findings in children and young adults with *Helicobacter pylori i*nfection. *Yonsei Medical Journal*.

[28] Harris P. R., Wright S. W., Serrano C. (2008). *Helicobacter pylori* gastritis in children is associated with a regulatory T-cell response. *Gastroenterology*.

